# A New Tool for Detecting COVID-19 Psychological Burden Among Postacute and Long-term Care Residents (Mood-5 Scale): Observational Study

**DOI:** 10.2196/26340

**Published:** 2021-03-10

**Authors:** William E Mansbach, Ryan A Mace, Melissa A Tanner

**Affiliations:** 1 Mansbach Health Tools, LLC Simpsonville, MD United States

**Keywords:** nursing homes, long-term care, COVID-19, depression, stress, coping, burden, mental health, elderly, older adults, risk, telehealth, self-assessment, scale, mood

## Abstract

**Background:**

Older adults are at high risk for developing serious somatic and psychological symptoms associated with COVID-19. Currently available instruments may not be sensitive to the concerns about COVID-19 in postacute and long-term care and their applications in telehealth remain to be clarified.

**Objective:**

We investigated the psychometric properties of the Mood-5 Scale (M5) as a rapid self-assessment of the COVID-19 psychological burden among postacute and long-term care residents.

**Methods:**

Residents (N=131), aged 50 years and above, from 20 postacute and long-term care facilities in Maryland, USA, were evaluated in-person or via telehealth (43/131, 32.8%) across a 4-week period (May 11 to June 5, 2020) during the COVID-19 pandemic. The COVID-19 psychological burden experienced by the residents was rated by geriatric psychologists who independently reviewed their clinical documentation. Psychometric analyses were performed on the M5 in relation to psychological tests, COVID-19 psychological burden, and diagnostic data collected during the evaluation.

**Results:**

The M5 demonstrated acceptable internal consistency (Cronbach α=.77). M5 scores were not confounded by demographic variables or telehealth administration (*P*>.08). Convergent validity for the M5 was established via positive associations with anxiety (*r*=0.56, *P*<.001) and depressive (*r*=0.49, *P*<.001) symptoms. An M5 cutoff score of 3 demonstrated strong sensitivity (0.92) and adequate specificity (0.75) for identifying COVID-19 psychological distress among postacute and long-term care residents (area under the curve of 0.89, positive predictive value=0.79, negative predictive value=0.91).

**Conclusions:**

The M5 is a reliable and valid tool for self-assessment of mood that can help identify postacute and long-term care residents with significant psychological burden associated with COVID-19. It can be completed in less than 1 minute and is appropriate for use in both in-person and virtual visits.

## Introduction

The base rates of depression and anxiety are high among postacute and long-term care (PA/LTC) residents. Approximately one-third of all residents in PA/LTC facilities experience significant depressive symptoms [1,2], whereas an estimated 5%-10% experience anxiety-related disorders [3,4]. These numbers are significantly higher among residents referred for neurocognitive evaluations. For instance, in a sample of PA/LTC residents referred for evaluation of mood and/or cognitive symptoms, 55% met the criteria for a major depressive episode, and 36.6% met the criteria for generalized anxiety disorder [5]. Although we found no studies investigating the psychological burden associated with COVID-19 in PA/LTC settings, there is evidence suggesting that the pandemic has contributed to an increase in mental health concerns. In a community sample, the American Psychiatric Association found that 36% of Americans reported that COVID-19 has had a significant impact on their mental health, and 48% reported feeling anxious about potentially contracting the infection [6]. The Centers for Disease Control and Prevention has issued a warning that people over the age of 65 years, those with serious underlying medical conditions [7,8], and those living in residential care settings are at the highest risk for developing severe illness from COVID-19; therefore, it is expected that these groups experience an increased psychological burden, placing them at a considerable risk of the development or exacerbation of psychiatric symptoms.

To our knowledge, evidence supporting a rapid mood screening tool that can be used to capture psychological burden associated with COVID-19 is currently lacking. Rapid screening is especially critical in the context of COVID-19. Under normal circumstances, health care providers are highly limited for the time that they can spend on assessments, particularly for co-occurring medical conditions [9,10]. Time is even more limited when the duration and extent of face-to-face encounters is capped to prevent the spread of infection, and competition for resources restricts the duration of virtual visits. This almost rules out the use of multiple measures or instruments that require more than 2 minutes of the providers’ time to administer. The measures of depression and anxiety that are most commonly used in PA/LTC settings, including the Patient Health Questionnaire (PHQ-9) [11] and the Generalized Anxiety Disorder 7-item (GAD-7) scale [12], assess depression or anxiety but not both. Moreover, formal anxiety screening is not required in PA/LTC settings. The Geriatric Depression Scale–Short Form [13] is a mood instrument developed specifically for older adults, but it has limited psychometric properties and double the number of items in the PHQ-9 [14].

In this study, we developed and validated the Mood-5 Scale (M5) to address barriers to its practical use in the context of COVID-19 by minimizing administration time, allowing for self-administration, and combining the assessment of depression and anxiety. The scale is an adapted version of the Brief Anxiety and Depression Scale (BADS), a screening tool that assesses both depressive and anxiety symptoms and is widely used by health care professionals in PA/LTC settings [5]. The M5 was designed so that it can be (1) self-administered by residents in PA/LTC settings for a variety of conditions, ranging from normal cognitive functioning to mild dementia; (2) completed in less than 1 minute; and (3) completed as part of an in-person or telehealth visit, which is particularly relevant in the context of the COVID-19 pandemic. We hypothesized that the M5 will be able to rapidly identify COVID-19–associated psychological burden, as well as clinical anxiety and depression.

## Methods

### Participants and Procedures

Residents (N=131) aged 50 years and above from 20 PA/LTC facilities in Maryland, USA, were evaluated by a behavioral health interdisciplinary team comprising 10 psychologists, 1 psychiatrist, and 10 nurse practitioners via in-person or telehealth visits. Data were collected across a 4-week period during the COVID-19 pandemic (ie, May 11 to June 6, 2020) to obtain a *snapshot* of the possible psychological burden during the pandemic, with the intention of sharing actionable information with the providers who care for PA/LTC residents. The relatively small sample size reflects the effort to fast-track the research process and maximize impact in the context of the current COVID-19 pandemic. Institutional approval was obtained from each PA/LTC facility, and all residents or their responsible parties completed a consent agreement. Furthermore, all residents were deidentified for the analysis. M5 items were derived from the standard evaluation procedures so that the residents experienced no additional burden through its administration. A battery of psychological tests, including the M5, BADS [5], and the Brief Cognitive Assessment Tool (BCAT) [15], was administered as part of the usual evaluation. The International Classification of Diseases 10th Revision (ICD-10) [16] and Clinical Dementia Rating Scale [17] were used to assign psychiatric diagnoses and dementia stages, respectively. Residents were excluded from the study analyses if they had incomplete M5 data, moderate-to-severe dementia, or were aged below 50 years. Demographic and clinical characteristics of the study sample are presented in Table 1.

**Table 1:** Select demographics and clinical characteristics (N=131).

Variable		Value, n (%)
Age (years), mean (SD)		76.12 (11.05)
**Gender**		
	Female	69 (52.67)
	Male	62 (47.33)
**Race**		
	White	110 (83.97)
	Black	14 (10.69)
	Other	4 (3.05)
	Missing	3 (2.29)
**Marital status**		
	Single	23 (17.56)
	Married	14 (10.69)
	Widowed	52 (39.69)
	Separated	6 (4.58)
	Divorced	34 (25.95)
	Missing	2 (1.53)
**Education (years completed)**		
	≤11	18 (13.74)
	12	55 (41.98)
	13-15	22 (16.79)
	16	17 (12.98)
	≥17	14 (10.69)
	Missing	5 (3.82)
**Facility**		
	Skilled nursing	87 (66.41)
	Assisted living	44 (33.59)
**Cognitive level**		
	No dementia	10 (7.63)
	MCI^a^	67 (51.15)
	Mild dementia	54 (41.12)
Telehealth delivery		43 (31.82)
COVID-19 distress		67 (51.15)

^a^MCI: mild cognitive impairment.

### Measures

#### Development of the M5

The M5 was adapted from BADS, which was chosen because it measures depression and anxiety factors separately, is used widely in PA/LTC settings, and has strong psychometric properties. Two items each from the depression and anxiety factors of BADS were selected for inclusion in the M5. A fifth item was added to address somatic or cognitive features. A panel of experts comprising 3 geriatric psychologists, 1 psychiatrist, and 2 PA/LTC medical directors vetted the instrument before data collection.

For standardized administration, residents were instructed as follows: “Think about how you have been feeling during the past month as you answer the following five questions. Please answer: ‘no’=0, ‘somewhat’=1, or ‘yes’=2.” The M5 items were written as follows:

1. Have you lost interest in activities that you had found pleasurable?
2. Do you worry about things more than usual?
3. For at least two consecutive days, have you felt depressed, hopeless, or down?
4. Are you feeling nervous, anxious, or “wound up” much of the time?
5. Are you experiencing fatigue, headaches, stomach upset, or memory problems?

Multimedia Appendix 1 presents the M5 items and standardized scoring instructions.

#### COVID-19 Psychological Burden

The outcome binary variable “COVID-19 psychological burden” was based on a geriatric psychologist’s independent review of the behavioral health providers’ clinical documentation. While completing the medical records of PA/LTC residents, health care professionals were required to directly ask the patient whether they were experiencing psychological symptoms *associated* with fear of contracting COVID-19 and/or social distancing precautions to reduce disease transmission. An affirmative score was assigned if the documentation supported that the resident was queried about the COVID-19 psychological burden and the resident made direct statements about experiencing increased anxiety or depressive symptoms associated with COVID-19 or if the health care professional observed increased anxiety or depression associated with COVID-19.

#### Validity Measures

The convergent and discriminant validity of the M5 were evaluated using the BADS and BCAT, respectively. The BADS is an 8-item mood questionnaire designed to identify anxiety and depression (score range 0-16) among older adults. The BCAT is a 21-item, multi-domain cognitive instrument (score range 0-21) that distinguishes among normal cognition, mild cognitive impairment, and dementia [15,18].

### Statistical Analysis

Analyses were performed in R (version 3.6.1; R Core Team) [19] using RStudio (version 1.2.5019; RStudio Team) software [20]. Descriptive statistics were used to report demographics, clinical characteristics, and study measures. Pearson correlations, independent sample *t* tests, and analysis of variance were performed to investigate the relationship between these variables and the M5. Cronbach α was used to estimate internal consistency. Receiver operator characteristic curve analyses examined the ability of the M5 to identify COVID-19 psychological burden. Despite the compressed data collection period, the sample size was sufficient for preliminary reliability [21,22]. Residents with missing study measures were removed pairwise to maximize the use of available M5 data.

## Results

### Preanalysis

Table 2 reports descriptive statistics for the M5 and validity measures. M5 scores were not associated with gender, race, marital status, education, or provider discipline (*P*>.05). Residents in skilled nursing settings (87/131, 66.4%) reported higher M5 scores than residents in assisted living settings (diff=1.73; 95% CI 0.29-3.18; *P*=.01). Younger age was associated with higher M5 scores (*r*=−0.19, *P*=.03). M5 scores did not differ as a function of telehealth (43/131, 32.8%) or in-person evaluations (diff=0.08; 95% CI −0.97 to 1.13; *P*=.88).

**Table 2:** Descriptive statistics of the various study measures used.

Study measure	n (%)	Mean (SD)	Minimum	Maximum	Skewness	Kurtosis
M5^a^	131 (100)	3.60 (2.86)	0	10	0.57	−0.59
BCAT^b^	70 (53.4)	34.61 (6.38)	22	46	−0.05	−1.02
BADS^c^ AF^d^	110 (83.9)	2.70 (1.78)	0	6	0.47	−0.75
BADS DF^e^	110 (83.9)	3.31 (3.01)	0	10	0.68	−0.59

^a^M5: Mood-5 Scale.

^b^BCAT: Brief Cognitive Assessment Tool.

^c^BADS: Brief Anxiety and Depression Scale.

^d^AF: Anxiety Factor of BADS.

^e^DF: Depression Factor of BADS.

### Psychometric Analyses

The M5 demonstrated acceptable internal consistency (Cronbach α=.77, 95% CI 0.71-0.83). Item-level statistics for the M5 are presented in Table S1 of Multimedia Appendix 1. Convergent validity for the M5 was established via positive and moderate associations with anxiety (*r*=0.56, *P*<.001) and depressive (*r*=0.49, *P*<.001) symptoms on the BADS (Table 3). Discriminant validity was confirmed for the M5 by negligible relationship with cognitive functions on the BCAT (*r*=0.17, *P*=0.15).

**Table 3:** Correlation analysis (Pearson r and two-tailed *P* values) among the study measures.

Measure		M5^a^	BCAT ^b^	BADS^c^ AF^d^	BADS DF^e^
**M5**					
	*r*	1	0.17	0.49	0.56
	*P* value	—^f^	.15	<.001	<.001
**BCAT**					
	*r*	0.17	1	0.03	0.23
	*P* value	.15	—	.81	.06
**BADS AF**					
	*r*	0.49	0.03	1	0.52
	*P* value	<.001	.81	—	<.001
**BADS DF**					
	*r*	0.56	0.23	0.52	1
	*P* value	<.001	.06	<.001	—

^a^M5: Mood-5 Scale.

^b^BCAT: Brief Cognitive Assessment Tool.

^c^BADS: Brief Anxiety and Depression Scale.

^d^AF: Anxiety Factor of BADS.

^e^DF: Depression Factor of BADS.

^f^Not applicable.

Residents with generalized anxiety disorder or anxiety disorder due to a known physiological condition reported significantly higher M5 scores (41/131, 31.3%) than the remaining residents without anxiety diagnoses (diff=1.94; 95% CI −0.92 to 2.95; t_129_=3.78; *P*<.001). The effect size for this difference was medium (Cohen *d=*0.71; 95% CI 0.33-1.09).

Residents with moderate or severe recurrent major depressive disorder (without psychotic symptoms) reported significantly higher M5 scores (22/131, 16.8%) than the remaining residents without these depression diagnoses (diff=3.65; 95% CI 2.49-4.82; t_129_=6.21; *P*<.001). The effect size for this difference was large (Cohen *d*=1.45; 95% CI 0.96-1.95).

### COVID-19 Psychological Distress

**Figure 1:**
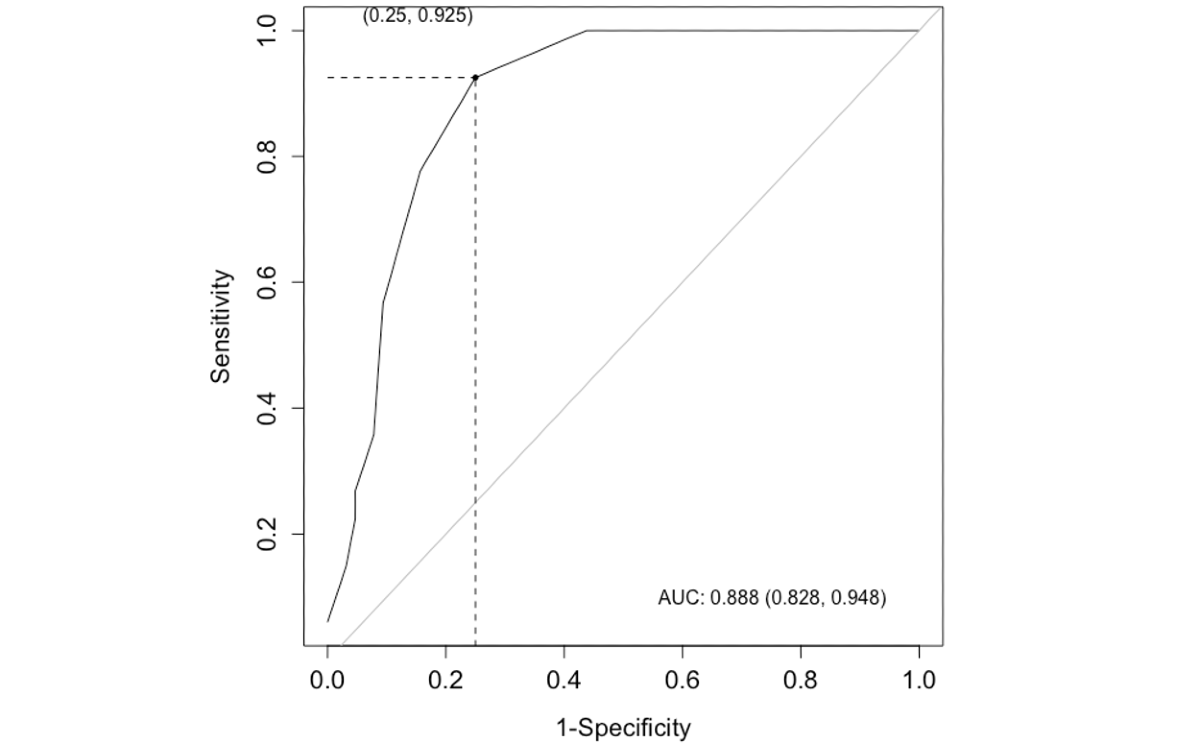
Receiver operating characteristics and area under the curve (AUC) were calculated from sensitivity and (1−specificity) values for the Mood-5 Scale for identifying COVID-19 psychological distress among older adults.

An M5 cutoff score of 3 (ie, scores ≥3) maximized the product of sensitivity (0.92) and specificity (0.75) for detecting COVID-19 psychological distress among PA/LTC residents (positive predictive value=0.79, negative predictive value=0.91). Area under the curve was 0.89 (95% CI 0.83-0.95), and 16% (21/131) of the residents were incorrectly classified (16 false positive and 5 false negative). Table 4 presents the properties for alternative M5 cutoff scores. Figure 1 illustrates the M5 receiver operative characteristic curve.

**Table 4:** Predictive utility of several cutoff scores for the Mood-5 Scale.

Cutoff score	Value (95% CI)			
	Sensitivity	Specificity	PPV^a^	NPV^b^
2	1.00 (0.93, 1.00)	0.56 (0.43-0.68)	0.71 (0.60-0.79)	1.00 (0.88-1.00)
3	*0.93 (0.83-0.97)^c^*	*0.75 (0.62-0.85)*	*0.79 (0.69-0.87)*	*0.91 (0.79-0.96)*
4	0.78 (0.65-0.87)	0.84 (0.73-0.92)	0.84 (0.72-0.92)	0.78 (.66-0.87)

^a^PPV: positive predictive value.

^b^NPV: negative predictive value.

^c^Italicized values in the table indicate the M5 cutoff scores with the optimal product of sensitivity, specificity, positive predictive value, and negative predictive value for identifying COVID-19 psychological distress.

## Discussion

Our findings support the reliability and validity of the M5 as a mood scale that can identify PA/LTC residents with COVID-19 psychological burden. The M5 is a reliable and valid mood scale that can be completed rapidly, is appropriate for in-person or virtual visits, and can be self-administered. It can be facilitated by a staff member or completed by a resident prior to or during a visit with a health care professional. Given its brevity, the M5 fits easily into an attending physician’s assessment toolbox and can provide real-time information to guide the management of psychiatric medications. This may help rightsize psychotropic use, especially for PA/LTC settings wherein behavioral health specialists are lacking. We recommend a cutoff score of 3 (ie, scores ≥3) to identify those residents who are more psychologically vulnerable and may benefit from a formal mood evaluation. We selected a M5 cutoff score that emphasized sensitivity to identify residents who would benefit from specific counseling to address concerns about COVID-19. Such concerns could be associated with contracting the infection, reduced opportunities for meaningful engagement due to social distancing, and concerns about the health of loved ones.

Our study has several strengths, including (1) our use of the ICD-10 and the Clinical Dementia Rating scale for diagnoses, (2) determination of the COVID-19 psychological burden by an independent review performed by a geriatric psychologist, (3) feedback on the M5 from attending physicians and medical directors, and (4) a selection of widely used validity measures (eg, BCAT and BADS) developed specifically for PA/LTC settings. Owing to the urgency to develop a scale that could be applied to PA/LTC residents during the current COVID-19 pandemic, our sample size was relatively small. This is partly mitigated by the inclusion of residents from multiple settings. The next steps for our study should involve cross-validation, collecting additional data to investigate psychological burden over time as the prevalence of confirmed COVID-19 cases decline, and investigating the psychological burden and associated M5 scores assigned by health care professionals and staff who care for PA/LTC residents. The primary focus of this study was to establish a clinically relevant cutoff score for the M5. Future studies should compare the psychometric properties of the M5 to separate measures of anxiety and depression commonly used in PA/LTC settings, such as the GAD-7 and PHQ-9.

The most immediate implication of this study is that widespread deployment of the M5 in PA/LTC settings can identify residents who are at a higher risk for experiencing COVID-19–related psychological burden and facilitate timely intervention. However, the M5 has potential utility beyond its ability to identify residents with an increased psychological burden associated with COVID-19. For nursing homes, incorporating the M5 into standard screening practices would redress a shortcoming in the current Minimum Data Set (MDS 3.0), which mandates a depression screening but does not include an instrument sensitive to anxiety symptoms. The M5 is sensitive to *both* depressive and anxiety symptoms. The use of instruments that are sensitive to both anxiety and depression could help reduce rehospitalizations [23], thereby improving some quality measures. Finally, use of the M5 during postacute care can provide a mood baseline that can be used to track mood symptoms postdischarge, thus improving care transitions.

## Appendix

Multimedia Appendix 1 Item-level statistics for the M5.

## Abbreviations

BADS: Brief Anxiety and Depression Scale
BCAT: Brief Cognitive Assessment Tool
GAD-7: Generalized Anxiety Disorder 7-item
ICD-10: International Classification of Diseases 10th Revision
M5: Mood-5 Scale
PA/LTC: Post-acute and long-term care
PHQ-9: Patient Health Questionnaire
