# Supplementary material for: A New Tool for Detecting COVID-19 Psychological Burden Among Postacute and Long-term Care Residents (Mood-5 Scale): Observational Study
Source: JMIR Aging. 2021 Mar 10;4(1):e26340. doi: 10.2196/26340 (PMC8081160; doi:10.2196/26340)
Supplement: Multimedia Appendix 1 [file aging_v4i1e26340_app1.docx]

**Table S1.** Item-level statistics for the M5.

| *M5 item* | *M* | *SD* | *Skew* | *Kurtosis* | *Alpha* | *Inter-item* |  | *Item-total* |
| --- | --- | --- | --- | --- | --- | --- | --- | --- |
| 1. Anhedonia | 0.52 | 0.74 | 1.02 | -0.45 | 0.76 | 0.43 |  | 0.65 |
| 2. Excessive worry | 0.86 | 0.83 | 0.26 | -1.51 | 0.73 | 0.40 |  | 0.74 |
| 3. Depressed mood | 0.81 | 0.84 | 0.37 | -1.50 | 0.70 | 0.36 |  | 0.79 |
| 4. Irritability/agitation | 0.66 | 0.80 | 0.67 | -1.13 | 0.69 | 0.36 |  | 0.79 |
| 5. Somatic symptoms | 0.74 | 0.74 | 0.45 | -1.08 | 0.76 | 0.44 |  | 0.63 |

*Note.* Mood-5 Scale (M5). Alpha = Cronbach’s alpha if item deleted, inter-item = average inter-item correlation, item-total = correlation with total M5 score. Standardized scoring instructions: *“Think about how you have been feeling during the past month as you answer the following five questions. Please answer: ‘no’ = 0, ‘somewhat’ = 1, or ‘yes’ = 2.”*
